# Resveratrol attenuates oxidative injury in human umbilical vein endothelial cells through regulating mitochondrial fusion via TyrRS-PARP1 pathway

**DOI:** 10.1186/s12986-019-0338-7

**Published:** 2019-01-30

**Authors:** Jining Yang, Xi Zhou, Xianglong Zeng, Ou Hu, Long Yi, Mantian Mi

**Affiliations:** Research Center for Nutrition and Food Safety, Chongqing Key Laboratory of Nutrition and Food Safety, Institute of Military Preventive Medicine, Third Military Medical University, 30th Gaotanyan Main Street, Shapingba District, Chongqing, 400038 People’s Republic of China

**Keywords:** Endothelial cells, Atherosclerosis, Resveratrol, Mitochondrial dynamics, TyrRS, PARP1

## Abstract

**Background/aims:**

Oxidative stress-induced damage in endothelial cells is a crucial initiator of atherosclerosis (AS), which is highly related to excessive reactive oxygen species (ROS) and mitochondrial dynamics. Resveratrol (RSV) exerts beneficial effects against endothelial oxidative injury, while the underlying mechanisms have not been fully elucidated. Thus, we aimed to explore the role of mitochondria dynamics during the anti-oxidative activities of RSV in palmitic acid (PA)-stimulated human umbilical vein endothelial cells (HUVECs) and to verify whether tyrosyl transfer- RNA synthetase (TyrRS) and poly (ADP-ribose) polymerase 1 (PARP1) are targeted during this process.

**Methods:**

HUVECs were exposed to 200 μM of PA for 16 h before treated with 10 μM of RSV for 8 h. Cell viability was detected using Cell counting kit-8 (CCK-8) assay. The intracellular ROS level and mitochondria membrane potential (MMP) were measured using microplate reader and flow cytometry. The malondialdehyde and superoxide dismutase were measured using the microplate reader. The mitochondrial morphology and fusion process was observed under transmission electron microscopy and confocal microscopy. TyrRS and PARP1 were knocked down with the specific small interference RNAs (siRNA), and the protein expressions of TyrRS, PARP1, and mitochondrial fusion proteins (MFN1, MFN2, and OPA1) were measured by western blot.

**Results:**

RSV treatment suppressed the PA-induced injuries in HUVECs, including the damage to cell viability, oxidative stress, and loss of MMP. Additionally, RSV improved the protein levels of MFN1, MFN2, and OPA1 as well as inhibited the PA-induced fragmentation of mitochondria. However, the effects of RSV on oxidative stress and mitochondrial fusion were abolished by the pretreatment of siRNAs of TyrRS and PARP1, indicating that these effects of RSV were dependent on the TyrRS-PARP1 pathway.

**Conclusions:**

RSV attenuated endothelial oxidative injury by regulating mitochondrial fusion via TyrRS-PARP1 signaling pathway.

**Electronic supplementary material:**

The online version of this article (10.1186/s12986-019-0338-7) contains supplementary material, which is available to authorized users.

## Background

Atherosclerosis (AS) is a chronic disease characterized by the accumulation of lipids and fibrous plaques in the large arteries, which leads to hardening and narrowing of the arteries and causes most cardiovascular diseases (CVDs) [1]. Oxidative stress, the excessive production of reactive oxygen species (ROS), plays a vital role in the initiation and propagation of AS [2, 3]. The endothelial wall is the first barrier to prevent the oxidative stress damage. Thus, it is imperative to protect the endothelial cells in the prevention of AS.

Mitochondria, the major ROS generation sites, are motile organelles that are present in the mammalian cell and display a continuous cycle of fission and fusion, known as mitochondrial dynamics [4]. These mitochondrial dynamics are mediated by extensive protein machinery and, in combination with mitochondrial swelling/shrinking and removal of damaged mitochondria by mitophagy, determine net mitochondrial morphology. Mitochondrial fusion occurs by sequential merging of the mitochondrial outer membrane (MOM) and mitochondrial inner membrane (MIM) [5], which is ATP-dependent, requires a sufficiently negative trans-MIM electrical potential, and is mediated by two mitofusins (MFN1/MFN2; MOM-fusion) and the optic atrophy 1 protein (OPA1; MIM-fusion) [6–10]. During the last decade, accumulating evidence suggests that cellular and mitochondrial redox homeostasis is linked to mitochondrial dynamics, mediated by extensive protein machinery and determining net mitochondrial morphology [11].

For primary prevention of AS in early life, lifestyle modifications, mainly nutritional intervention without pharmacological treatment, would be an optimal strategy [12]. Resveratrol (RSV), a natural polyphenolic compound, is widely existing in fruits, such as grapes [13]. Epidemiological studies indicate that the Mediterranean diet, which is rich in RSV, is associated with a reduced risk of AS [14]. Our previous studies found that RSV could attenuate endothelial inflammation by inducing autophagy, as well as regulate mitochondrial ROS homeostasis, which is closely related to mitochondrial dynamics, thus reducing oxidative stress in endothelial cells. A recent study found that RSV might affect the activity of tyrosyl transfer-RNA synthetase (TyrRS), thereby stimulating poly(ADP-ribose) polymerase 1 (PARP1) [15]. However, whether RSV could regulate the mitochondrial dynamics through TyrRS-PARP1 signaling in endothelial cells is not fully elucidated. Thus, we hypothesized that RSV attenuates the oxidative stress by regulating mitochondrial fusion via TyrRS-PARP1 pathway in vascular endothelial cells.

To test the hypothesis, we used palmitic acid (PA), a type of saturated fatty acid, to establish an oxidative stress model in human umbilical vein endothelial cells (HUVECs). We found that in this oxidative stress model, RSV could improve the cell viability, reduce intracellular ROS level, preserve mitochondrial membrane potential (MMP), maintain the mitochondrial morphology in a tubular shape by regulating mitochondrial fusion proteins through TyrRS-PARP1 pathway. These results elucidated a new mechanism of protective effects of RSV on protecting endothelial cells, as well as broadened the possible targets to treat AS.

## Methods

### Cell culture and treatment

**Fig. 1:**
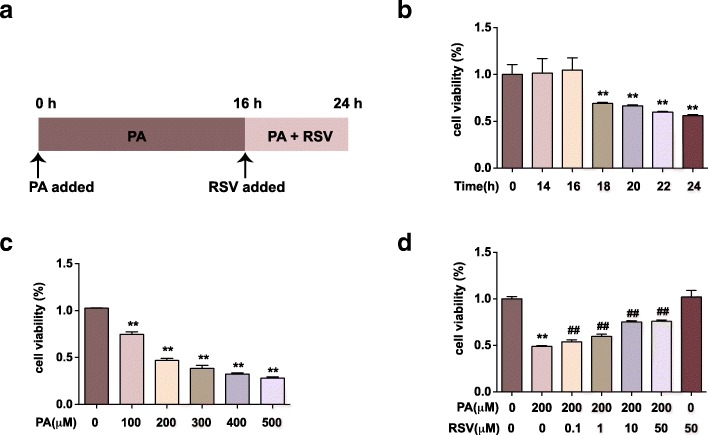
RSV promotes cell viability in PA-treated HUVECs. **a**: Cell treatment scheme for the major experiment. Cells were treated with 200 μM of PA for 16 h, and then RSV of specific concentration was added for 8 h. Cell viability was measured by CCK-8 assay and calculated from the ratio of the optical density of the experimental cells to that of the control cells (set as 100%). **b**: Cells were treated with 200 μM PA for different periods (14, 16, 18, 20, 22 and 24 h). **c:** Cells were treated with PA of different concentrations (100, 200, 300, 400 and 500 μM) for 24 h. **d**: Cells were treated as described in **a.** RSV of different concentrations (0.1, 1, 10 and 50 μM) were used. Values are expressed as means ± SD (*n* = 3); ^*^p < 0.05, ^**^p < 0.01 vs. the vehicle-treated control group; ^#^*p* < 0.05, ^##^*p* < 0.01 vs. PA-treated group

HUVECs were isolated as described previously [16]. This study was approved by the ethics committee of Army Medical University, and all the involved patients attained consent. HUVECs were cultured in M199 medium with 10% FBS (Hyclone, GE Healthcare Life Sciences, US), 1% penicillin-streptomycin (Thermofisher, US) and 0.1% vascular endothelial growth factor (Thermofisher, US) at 37 °C and 5% CO_2_. Cells from 3rd to 6th passages were used for all the following experiments. The primary cell treatment scheme was as follows: when cells grew to 80% confluence, PA (200 μM) was added for 16 h, followed by RSV (10 μM) treatment in the presence or absence of PA for an additional 8 h (Fig. 1a). As for the control group: the vehicle that dissolved PA (bovine serum albumin, BSA) was added for 16 h, followed by the vehicle that dissolved RSV (dimethyl sulfoxide, DMSO) for an additional 8 h.

### Cell counting kit–8 (CCK–8) assay

CCK-8 (Dojindo, Kumamoto, Japan) was used to detect cell viability. Briefly, 8 × 10^3^ cells were seeded into 96-well plates and then exposed to PA of different concentrations (0, 100, 200, 300, 400, 500 μM) for 24 h, or to PA of 200 μM for different time periods (0, 14, 16, 18, 20, 22, 24 h). In the detection of the effect of RSV on PA-induced damage, RSV of different concentrations (0.1, 1, 10, 50 μM) was added, followed by the exposure to PA for 16 h. Cells were co-cultured with RSV and PA for another 8 h. And then, 20 μL of CCK-8 was added to each well for 1 h at 37 °C. Absorbance at 450 nm was recorded by the microplate reader (Molecular Devices, Sunnyvale, CA, USA). The ratio of the optical density of the experimental cells to that of the control cells (set as 100%) was the cell viability.

### Intracellular ROS measurement

2′,7′- dichlorofluorescein diacetate (DCFH-DA, Beyotime Biotechnology, China) was used to label the intracellular ROS. After the indicated treatment, cells in 96-well plate were loaded with DCFH-DA (1:1000) in FBS-free M199 medium and incubated at 37 °C for 30 min. Cells were washed thrice with PBS, and the fluorescence intensity was detected with the emission wavelength of 525 nm and the excitation wavelength of 488 nm. The intracellular ROS in each group was quantified after the normalization by the corresponding cell viability. Meanwhile, we used flow cytometry (FCM) to detect the intracellular ROS. After treatment, cells in 6-well plates were collected by trypsin, incubated with DCFH-DA probe for 30 min at 37 °C and washed twice in PBS. The samples were then subjected to the flow cytometry (BD Biosciences, San Jose, CA) analysis as described previously [17].

### MMP measurement

JC-1 (Beyotime Biotechnology, China), a dye that can selectively enter into mitochondria and reversibly change color from green to red as the MMP increase, was used to detect MMP [18]. After the cell treatment as described above, cells were loaded with JC-1 and incubated at 37 °C for 30 min. Next, cells were washed thrice by PBS. We detected the fluorescence intensity with the emission wavelength of 525 nm and the excitation wavelength of 488 nm (green fluorescence) as well as the emission wavelength of 590 nm and the excitation wavelength of 525 nm (red fluorescence), respectively. Fluorescence intensity ratio was calculated to reflect the MMP. Meanwhile, we also used FCM (BD Biosciences, San Jose, CA) to detect the MMP [19].

### Detection of malondialdehyde (MDA) and superoxide dismutase (SOD)

We used the Lipid Peroxidation MDA assay kit (Beyotime Biotechnology, China) to detect the MDA levels both in the cell culture medium and cell lysates. We used Total Superoxide Dismutase Assay Kit with WST-8 (Beyotime Biotechnology, China) to detect the SOD activity in cell lysates. After the indicated treatment as described above, cell lysates were prepared, and the protein concentration was measured using the BCA assay (Beyotime Biotechnology, China) [20]. The MDA and SOD levels were quantified according to the manufacturer’s instructions.

### Transmission electron microscopy (TEM)

Cells were harvested and fixed in glutaraldehyde for 24 h, post-fixed with 1% OsO4 for 1.5 h, washed, and stained in aqueous uranyl acetate for 1 h. The samples were then rewashed, dehydrated with the graded alcohol, and embedded in Epon-Araldite resin (Canemco & Marivac, Lakefield, Quebec, Canada). Ultrathin sections were obtained by an ultramicrotome (Reichert-Jung, Inc., Cambridge, UK), counterstained with 0.3% lead citrate, and visualized on a transmission electron microscope (EM420, Koninklijke Philips Electronics N.V., Amsterdam, The Netherlands).

### Mitochondrial morphology tracing using MitoTracker

Cells were treated as described above and then loaded with MitoTracker® Red CMXRos (125 nM, Cell Signaling Technology, US) at 37 °C for 30 min to label the mitochondria. After washing three times by PBS, cells were imaged by laser scanning confocal microscope (Zeiss, Germany) under the excitation wavelength of 577 nm and the emission wavelength of 590 nm. Percentage of cells without elongated mitochondria in each group was calculated as reported previously [21].

### Small interference RNA (siRNA) assay

For RNA interference experiments, cells were treated with the targeted siRNAs (Santa Cruz Biotechnology, US, sequence information: Additional file 1) or the corresponding control siRNA using Lipofectamine 2000 (Thermofisher, US) mix in Opti-MEM Reduced Serum Medium (Thermofisher, US) for 24 h as described in the manufacturer’s instructions. Then, cells were treated as indicated above. The transfection efficiency of each siRNA was detected by quantitative real-time polymerase chain reaction (qRT-PCR).

### RNA extraction and qRT-PCR

Cells were harvested in RNAiso Plus reagent (Takara Bio, Japan), and total RNA was extracted according to the manufacturer’s instructions. RNA concentration and purity were measured using a NanoDrop 2000 spectrophotometer (Thermo Scientific, USA). The first-strand cDNA was synthesized by reverse transcription with random primers using a PrimeScript RT Master Mix (Takara Bio, Japan). Quantitative real-time PCR was carried out with the qTower 2.2 real-time PCR system (Analytik Jena, Germany) using SYBR Premix Ex Taq II (Tli RNaseH Plus) (Takara Bio, Japan). The primers for the targeted genes were synthesized by Sangon Biotech (Shanghai, China). The primer sequences used for gene expression analysis are in Additional file 2. The amplification profile consisted of denaturation at 95 °C for 30 s, followed by 40 cycles of 95 °C for 5 s and 60 °C for 30 s. Relative fold-changes in gene expression were analyzed by the 2^−ΔΔCt^ method and normalized to the internal control gene β-actin (ACTB).

### Western blotting

The whole cell extracts were obtained by cell lysis buffer (Cell Signaling Technology, USA) with 0.5% protease inhibitor cocktail (Sigma, USA) and 1% phosphatase inhibitor cocktail I (Sigma, USA). Briefly, Equal amounts (30 μg) of proteins were resolved by 10% sodium dodecyl sulfate-polyacrylamide gel electrophoresis (SDS-PAGE) and then electroblotted onto polyvinylidene difluoride membranes for western blot analysis. Blots were probed with primary antibodies overnight at 4 °C. The primary antibodies applied were listed in Additional file 3. Membranes were incubated with a secondary antibody (1:5000), and the immunostained bands were visualized with Immobilon Western Chemiluminescent HRP Substrate (Millipore, USA). The signal was captured by Fusion FX (Vilber Lourmat, France). Densitometry analysis was computed using ImageJ software [22].

### Statistical analysis

Data analysis was performed with SPSS 19.0 software (Chicago, USA). All experimental data were expressed as the mean ± SD. Statistical differences among groups were determined with either Student’s t-test (for two groups) or one-way analysis of variance (ANOVA) followed by LSD post hoc tests (for multiple group comparisons). *P* values less than 0.05 were considered statistically significant. Each experiment has performed a minimum of 3 times.

## Results

### RSV promotes cell viability in PA-treated HUVECs

PA is a type of saturated fatty acid, which is usually used to establish endothelial injury model in vitro [23]. We used PA of different concentrations to treat HUVECs for 24 h and found that the cell viability reduced in a dose-dependent way (Fig. 1b). At the concentration of 200 μM, the cell viability declined to (46.9 ± 1.88) % compared with the control group (*p* < 0.01), which indicated that 200 μM was around the IC50 of PA to HUVECs. Then, we used the concentration of 200 μM PA to treat HUVECs for different periods (Fig. 1c). The cell viability started to decrease after 18 h of PA treatment and declined in a time-dependent manner (*p* < 0.01). In our previous studies, HUVECs was pretreated with RSV 2 h before the following exposure to PA treatment [16, 24]. Thus, we established our “RSV treating PA-injury model” by adding RSV of different concentrations to HUVECs after 16 h of PA treatment (Fig. 1a). We found that the decreased cell viability induced by PA treatment was notably ameliorated by different concentrations of RSV treatment (p < 0.01) (Fig. 1d). Moreover, 10 μM of RSV was used for the following study. These findings indicated that RSV could promote cell viability in PA-treated HUVECs.

### RSV attenuates PA-induced oxidative stress in HUVECs associated with TyrRS and PARP1

**Fig. 2:**
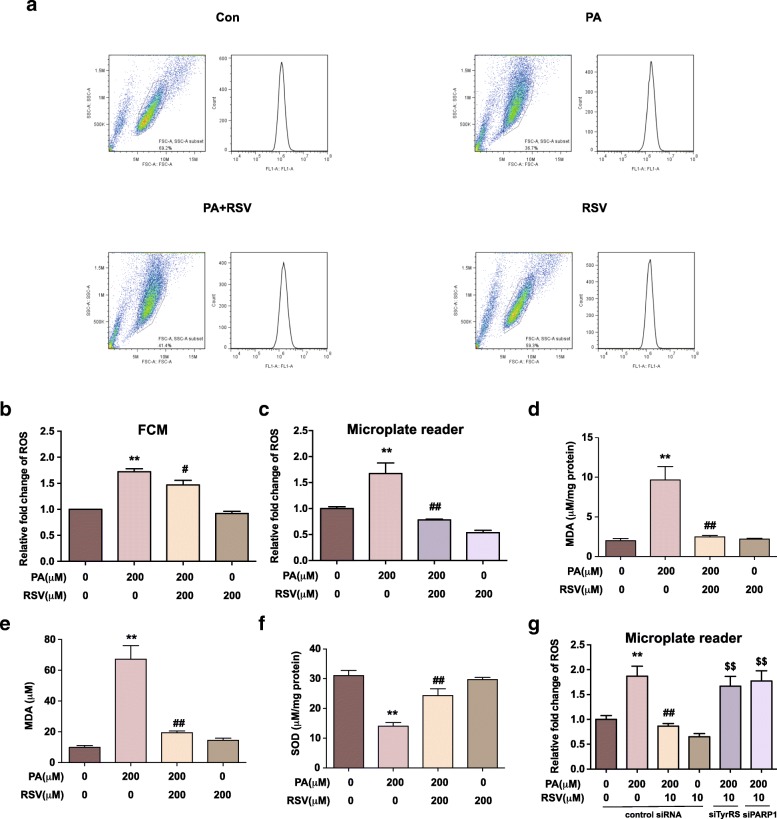
RSV attenuates PA-induced oxidative stress in HUVECs through TyrRS-PARP1 pathway. Cells were treated as indicated and labeled by DCFH-DA probe. **a-b:** Representative images (**a**) and quantification of intracellular ROS levels by FCM assay (**b**) **c**: Quantification of ROS levels by the microplate reader. **d-f**: Quantification of MDA of the medium (**d**) and cell lysates (**e**), the activity of SOD of the cell lysates (**f**). **g**: Cells were pretreated with siRNA of TyrRS, PAPR1, and the vehicle and were treated as indicated. The fluorescence intensity of cells labeled by DCFH-DA was measured by the microplate reader. Values are expressed as means ± SD (n = 3); ^*^p < 0.05, ^**^p < 0.01 vs. the vehicle-treated control group; ^#^*p* < 0.05, ^##^*p* < 0.01 vs. vehicle + PA-treated group; ^$^*p* < 0.05, ^$$^*p* < 0.01 vs. vehicle + PA + RSV-treated group

To elucidate the effects of RSV on PA-induced oxidative stress in HUVECs, we examined the intracellular ROS level in HUVECs. We labeled the intracellular ROS using a DCFH-DA probe and quantified it by FCM (Fig. 2a-b) and fluorescence microplate reader (Fig. 2c), respectively. In both of the assays, the ROS levels were significantly up-regulated in the PA-treated group with (172 ± 4) % by FCM assay (Fig. 2b) and (167 ± 17) % by the microplate reader (Fig. 2c) compared to the control group (*p* < 0.01). However, the increase of ROS induced by PA was notably suppressed by RSV treatment, with a decreasing rate of (15 ± 7) % in FCM assay and (53 ± 1.4) % in microplate reader assay (*p* < 0.05). The two assay both proved that RSV could suppress the intracellular ROS level in our model, whereas the variance between the two assays was mainly due to the different algorithms of fluorescence. Overall, these results indicated that RSV could attenuate PA-induced intracellular ROS in HUVECs.

MDA is a lipid peroxidation product [25], and SOD acts as the first line of defense against ROS [26]. Both of them are indicators of ROS-mediated injury. We found that PA induced a significant increase of MDA in the supernatants and cell lysates, which was inhibited by RSV treatment (*p* < 0.01) (Fig. 2d-e). Also, PA inhibited the SOD activity in HUVECs, but RSV suppressed the effect (p < 0.05) (Fig. 2f).

The previous study reported that TyrRS might be involved in the RSV’s biological functions, thus regulating PARP1 [27], which then interact with lots of other downstream genes on multi-aspects. Therefore, we hypothesized that TyrRS-PARP1 pathway might play a role in the anti-oxidative effects mediated by RSV in HUVECs. Therefore, we used siRNAs of TyrRS and PARP1 to knock down these genes to investigate our hypothesis. (The effect of RNA interference was shown in Additional file 4). After knocking down TyrRS and PARP1 by the corresponding siRNA, we detected the intracellular ROS level in HUVECs (Fig. 2g). We found that the anti-oxidative effect of RSV in HUVECs was abolished when TyrRS or PARP1 was knocked down (*p* < 0.01). These results suggested that RSV attenuate PA-induced oxidative stress in HUVECs through TyrRS and PARP1.

### RSV maintains MMP in PA-treated HUVECs associated with TyrRS and PARP1

**Fig. 3:**
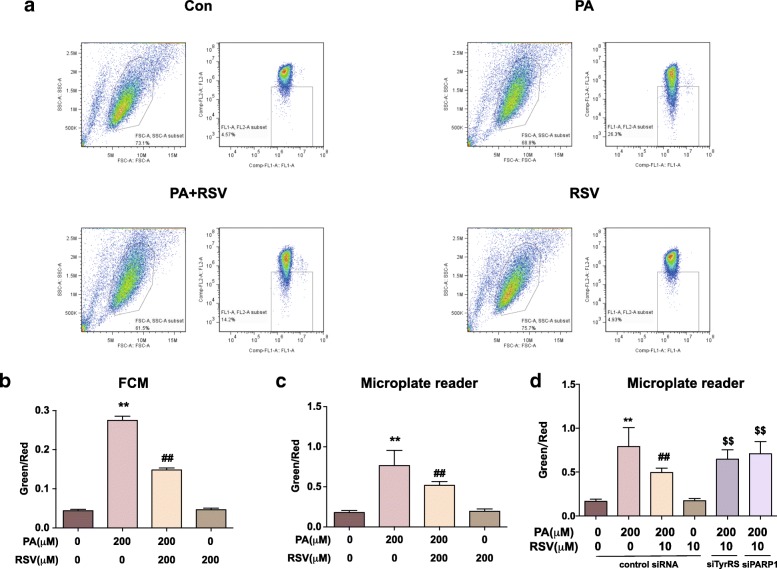
RSV maintains mitochondria function in PA-treated HUVECs through TyrRS-PARP1 pathway. Cells were treated with 200 μM PA for 16 h, co-cultured with 10 μM RSV for another 8 h and then labeled by JC-1 probe as described in the methods and materials section. **a-b**: Representative images (**a**) and quantification (**b**) of FCM on detecting red to green ratio of fluorescence intensity. **c:** Quantification of green to red ratio of fluorescence intensity by the microplate reader. **d**: Cells were pretreated with siRNA of the vehicle, TyrRS or PAPR1 for 24 h before cultured with 200 μM PA for 16 h and co-cultured with 10 μM RSV for another 8 h. Cells were then labeled by JC-1 probe. Quantification of green to red ratio of fluorescence intensity by microplate reader is displayed in the bar graph. Values are expressed as means ± SD (n = 3); ^*^p < 0.05, ^**^p < 0.01 vs. the vehicle-treated control group; ^#^*p* < 0.05, ^##^*p* < 0.01 vs. vehicle + PA-treated group; ^$^*p* < 0.05, ^$$^*p* < 0.01 vs. vehicle+PA + RSV-treated group

MMP is a primary indicator of mitochondria health, the loss of which usually correlates with the dysfunction of mitochondria and leads to cell death [26]. We labeled the MMP using JC-1 probe: the increased ratio of green to red fluorescence represents the loss of MMP. Then, we detected the ratio using FCM (Fig. 3a-b) and fluorescence microplate reader (Fig. 3c), respectively. PA led to an dominantly increased ratio of green to red by FCM assay (626 ± 20) % and microplate reader (432 ± 11) %, while RSV treatment suppressed the changes with (46 ± 2) % by FCM assay and (33 ± 6) % by the microplate reader (*p* < 0.01), respectively. Moreover, cells were pretreated with TyrRS and PARP1 siRNAs to study the role of TyrRS and PARP1 in the effects of RSV on MMP. As shown in Fig. 3d, the effect of RSV on maintaining MMP in PA-treated HUVECs was abolished by the knockdown of TyrRS and PARP1 (p < 0.01). Overall, RSV suppressed the PA-induced loss of MMP in HUVECs, which was associated with TyrRS and PARP1.

### RSV promotes mitochondrial fusion in PA-treated HUVECs associated with TyrRS and PARP1

**Fig. 4:**
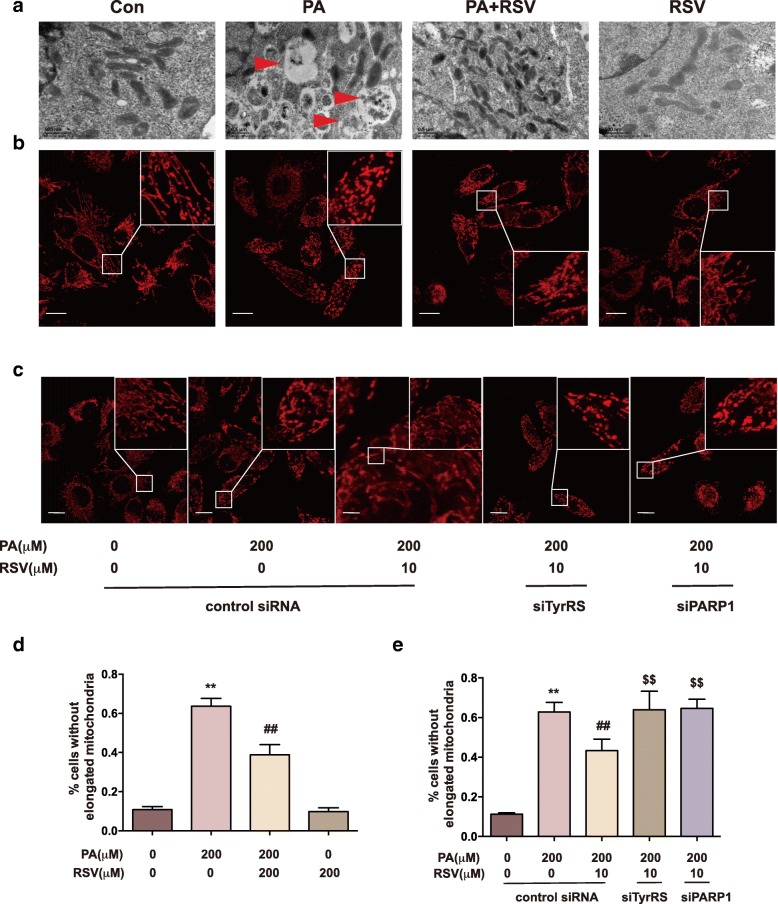
RSV preserves mitochondria in the tubular shape in PA-treated HUVECs through TyrRS-PARP1 pathway. Cells were treated with 200 μM PA for 16 h, co-cultured with 10 μM RSV for another 8 h. **a**: Representative TEM images of HUVECs after treatment as above. Arrows indicate the lipid droplets, scale bars: 500 nm. **b, d**: Cells after the treatment above were labeled with MitoTracker. Representative images(**b**) were acquired by a laser scanning confocal microscope; scale bars: 500 nm. The percentage of cells without elongated mitochondria in each group were calculated (**d**). **c, e**: Cells were pretreated with siRNA of the vehicle, TyrRS or PAPR1 for 24 h before cultured with 200 μM PA for 16 h and co-cultured with 10 μM RSV for another 8 h. Cells then were labeled with MitoTracker. Representative images (**c**) were acquired by the laser scanning confocal microscope; scale bars: 500 nm. The percentage of cells without elongated mitochondria in each group were calculated (**e**). Values are expressed as means ± SD (n = 3); ^*^p < 0.05, ^**^p < 0.01 vs. the vehicle-treated control group; ^#^*p* < 0.05, ^##^*p* < 0.01 vs. vehicle + PA-treated group; ^$^*p* < 0.05, ^$$^*p* < 0.01 vs. vehicle+PA + RSV-treated group

The morphology of mitochondria is closely related to the function and redox state of mitochondria [28]. Filamentous and tubular mitochondria are generally assumed to be healthy and in a well-functional state [28]. We observed the morphology of mitochondria in HUVECs using TEM as well as confocal microscopy. As the representative images shown in Fig. 4a, lots of lipid droplets, indicated by red arrows, entered the cells, leading to mitochondria crowded and malformed in shape. However, the RSV treated cells contained fewer droplets, and the morphology of mitochondria was similar to the control group. Then, we labeled mitochondria with Mitotracker and imaged them under the confocal microscope to detect the overall mitochondrial shape (Fig. 4b). We quantified the percentage of cells without elongated mitochondria in each group (Fig. 4d). In the PA-treated group, mitochondria were mostly in fragmented or spherical shape, suggesting malfunctioning of mitochondria. However, in the RSV-treated group, the morphology of mitochondria was much better improved into a tubular shape (*p* < 0.01). These results indicated that RSV could promote mitochondria to become the tubular shape, also known as fusion, thus alleviating damage. Also, we investigated whether TyrRS and PARP1 were involved in the mitochondrial morphology regulation by RSV treatment, by using TyrRS and PARP1 siRNAs. As shown in Fig. 4c and e, after TyrRS and PARP1 were knocked down, the effect that RSV promoted tubular mitochondria was inhibited. Accordingly, RSV could promote mitochondria fusion in PA-treated HUVECs associated with TyrRS and PARP1.

### RSV regulates mitochondria fusion proteins expressions through the TyrRS-PARP1 signaling pathway

**Fig. 5:**
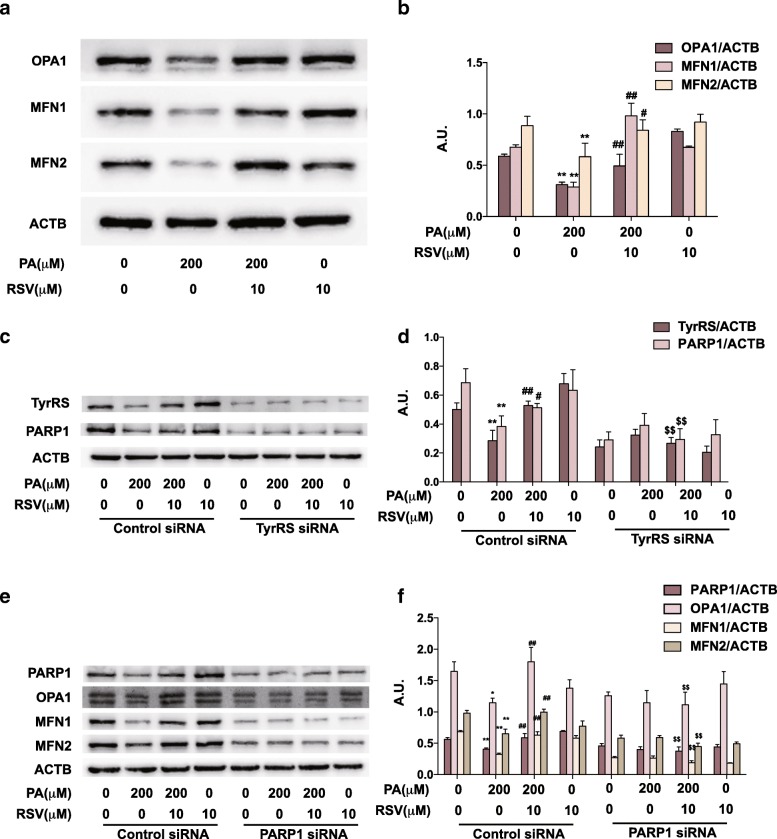
RSV regulates mitochondria fusion proteins through the TyrRS-PARP1 pathway. **a-b:** Cells were treated with 200 μM PA for 16 h, co-cultured with 10 μM RSV for another 8 h, and then proteins were extracted for western blot. The protein levels of OPA, MFN1, and MFN2 were analyzed by western blotting (**a**) and quantification displayed by bar graph (**b**). **c-d:** Cells were pretreated with siRNA of TyrRS (**c-d**) or PAPR1 (**e-f**) for 24 h before cultured with 200 μM PA for 16 h and co-cultured with 10 μM RSV for another 8 h. The protein levels of TyrRS, PARP1, OPA1, MFN1, and MFN2 were analyzed by western blotting (**c, e**) and quantification displayed by bar graph (**d, f**). Values are expressed as means ± SD (n = 3); ^*^p < 0.05, ^**^p < 0.01 vs. the vehicle-treated control group; ^#^*p* < 0.05, ^##^*p* < 0.01 vs. vehicle + PA-treated group; ^$^*p* < 0.05, ^$$^*p* < 0.01 vs. vehicle + PA + RSV-treated group. A.U., arbitrary units

The fusion of mitochondria is regulated by several critical proteins including OPA1, MFN1, and MFN2, the up-regulation of which could enhance mitochondrial fusion [29]. Thus, we first detected these protein levels in HUVECs by western blot (Fig. 5a-b). The fusion proteins (OPA1, MFN1, and MFN2) in HUVECs were significantly downregulated in PA-treated cells (*p* < 0.05), consistent with the less-tubular phenotype observed under microscopy (Fig. 4b, d). However, these proteins were up-regulated under the treatment of RSV (*p* < 0.05). These results proved that, on the protein level, RSV could help mitochondria preserve the tubular morphology when damaged by PA.

Then, we used siRNAs of TyrRS and PARP1 to knock down the corresponding genes. As shown in Fig. 5c-d, TyrRS and PARP1 were up-regulated in the RSV + PA-treated group, compared to the PA-treated group (*P* < 0.05), indicating that RSV could increase the expressions of TyrRS and PARP1 with the treatment of PA. However, the up-regulation of PARP1 by RSV treatment was inhibited when TyrRS was knocked down by the specific siRNA. Also, the up-regulation of fusion proteins (OPA1, MFN1, and MFN2) by RSV was suppressed when PARP1 was knocked down by the corresponding siRNA (Fig. 5e-f). These results suggested that RSV regulated mitochondria fusion proteins through the TyrRS-PARP1 pathway.

## Discussion

**Fig. 6:**
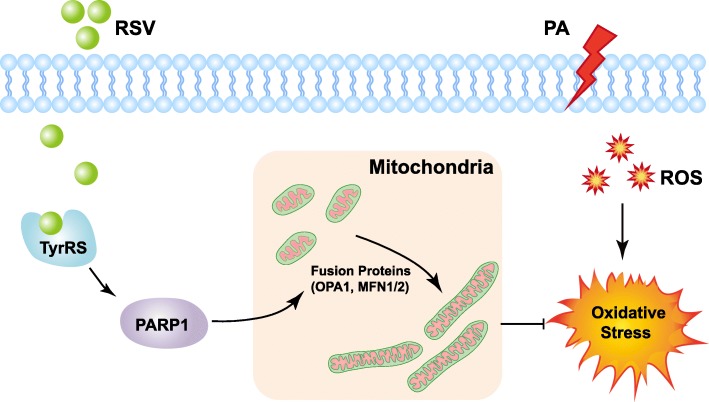
Proposed mechanism for RSV in the regulation of PA-induced oxidative stress in HUVECs. Under the stimulation of PA, HUVECs suffer from oxidative stress. In the treatment of RSV, TyrRS-PARP1 pathway is activated, which then promotes the expression of mitochondrial fusion proteins (OPA1, MFN1/2). The enhancing of mitochondrial fusion finally reduces the oxidative stress in HUVECs

In this study, we found, for the first time, that RSV could attenuate PA-induced endothelial oxidative stress through regulating mitochondrial fusion via the TyrRS-PARP1 signaling pathway (Fig. 6). CVDs are the number one cause of death [30], bringing a substantial economic burden globally [31]. Atherosclerosis, a disease in which plaque builds up inside the arteries, plays a central role in the development of CVDs [1]. ROS is a natural byproduct of the normal metabolism of oxygen [32]. However, the risk factors of AS, such as smoking, hypertension, hyperglycemia, consuming a diet high with saturated fat, obesity, or insulin resistance, can cause a robust increase of ROS [33], known as oxidative stress, leading to a cascade of events: oxidative modification of LDL, inflammation, cellular apoptosis and endothelium injury [15]. Among them, the ROS-induced endothelial injury is considered to be the initial factor of AS [15]. RSV, a kind of natural polyphenol compounds, is well-known for its antioxidant property and protective role in the cardiovascular system [34]. Cumulative studies have reported the effects and possible mechanisms of RSV on protecting endothelial cells by scavenging excess ROS [35–40]. However, the exact mechanisms remain to be elucidated.

In this study, we used PA to establish endothelial oxidative stress model to simulate the high saturated fatty acid diet in AS. In the most previous studies of the antioxidant effects of RSV, oxidative stress model is established by H_2_O_2_, which is a type of direct exogenous ROS [41–44]. However, although RSV can directly scavenge ROS in vitro, its antioxidant properties in vivo are more likely to be attributed to its effects as a gene regulator [34]. In the H_2_O_2_-induced oxidative stress model, attention is much paid on the effects of RSV on reducing the ROS on the chemical level. However, the biological regulations by RSV cannot be well detected. For example, it is hard to determine whether RSV can regulate the generation of ROS, mainly from mitochondria, in that model. In our study, we measured the ROS generation in PA-induced group by two methods: fluorescence microplate reader and flow cytometry (Fig. 2a-c). Both of the experiments have proved that PA can stimulate the ROS generation of HUVECs. Actually, PA has already been used to establish the AS model in human coronary artery endothelial cells [23]. Also, the oxidative stress stimulating property of PA in other types of cells have been proved in previous studies [23, 45, 46], which are consistent with our results. Thus, PA-treated HUVECs is a suitable model for studying the anti-oxidative mechanism of RSV.

Although PA is also a well-known chemical to induce insulin resistance in muscle system [47] and Parra et al. found insulin played a role in mitochondria fusion of cardiomyocytes [48], the characteristics and regulation of mitochondria in endothelial cells are entirely different from the muscle cells. Quintero et al. demonstrated that the primary role of mitochondria in vascular ECs might not be to generate ATP or consuming glucose in the blood as the muscle cells do, but to act as signaling organelles using ROS as signaling molecules [49]. Thus, the role of ROS stimulated by PA is of considerable importance in our model. However, it is still unclear whether insulin plays a role in endothelia cells, this would be an interesting question to be further studied.

In our study, we found that RSV can improve the viability of endothelial cells by regulating the mitochondrial morphology, maintaining the MMP and attenuating PA-induced ROS. Mitochondria are usually motile and continuously fuse and divide, which is recognized as mitochondrial dynamics [50]. In physiological conditions, mitochondria are tended to be tubular or filamentous and maintain healthy functions; but they change into fragmented or spherical in stressed conditions and become dysfunctional [50]. Recently, it is generally recognized that the three aspects: redox homeostasis, mitochondrial functional state, and mitochondrial morphology, interact with each other [11]. Excess ROS production can lead to a decrease of MMP, which is an initiator of cell death. On the other hand, the loss of MMP evokes the mitochondria generating excessive ROS and deteriorates the redox homeostasis [51]. Also, parallel changes in ROS levels and mitochondrial morphology have been reported in many experimental studies. For example, Koopman et al. found that in the patient primary fibroblasts with a significantly reduced CI activity displayed a fragmented mitochondrial phenotype along with highly increased ROS levels [52]. Moreover, the fusion proteins (MFN1, MFN2) can be regulated by exogenous H_2_O_2_, thus leading the mitochondrial fragmentation and dysfunction [53]. The reciprocal relations of the three aspects provide us with a hint that if we could find a way to change one aspect of this regulation circuit, the cell viability could be much improved. Here, in our study, we explored and demonstrated the possibility that RSV suppresses the PA-induced cell death by regulating the mitochondrial morphology, attenuate the ROS and improve mitochondrial function.

RSV has numerous targets to reduce oxidative stress [34]. The NAD + −dependent histone/protein deacetylase sirtuin 1(SIRT1) and nuclear factor-E2-related factor-2 (NRF2) are particularly important. RSV can activate SIRT1, thus up-regulate a range of antioxidant enzymes, such as SOD enzymes, glutathione peroxidase 1 (GPx1), and catalase [37–39]. Treatment of cells with resveratrol leads NRF2 to release from Kelch-like erythroid cap’n’collar homolog associated protein 1 and NRF2 translocation to the nucleus, thus up-regulating gene expression of antioxidant defense enzymes NAD(P)H:quinone oxidoreductase 1 and Haem oxygenase 1 [38]. Also, RSV can rapidly activate endothelial nitric oxide synthase (eNOS) by stimulating the membrane estrogen receptor, enchancing endothelial nitric oxide production [54, 55]. However, studies have been focused on these secondary targets until Sajish and Schimmel [27] found that TyrRS is the direct biological target of RSV. TyrRS is a homodimer of a 528-amino acid polypeptide and eukaryotic aminoacyl-tRNA synthetase known to contain an appended eukaryote-specific carboxy (C)-terminal endothelial monocyte–activating polypeptide II–like domain, acting as a general stress transducer [56]. Recently, it has been reported that RSV can promote nuclear translocation of endogenous TyrRS, concomitant with autoPARylation of PARP1, thus regulating a range of downstream genes [27]. Both TyrRS and PARP1 are considered being the critical stress transducers [27, 56, 57]. However, whether the downstream genes of TyrRS-PARP1 pathway include the mitochondrial fusion proteins is not known. In our present study, we found, for the first time, that the regulation of RSV on mitochondrial morphology, MMP and ROS generation is dependent on the TyrRS-PARP1 pathway, which highlights the importance and substantial basis for further studies on phytochemical therapy for AS.

Generally, our findings provided a novel mechanism in which RSV attenuates PA-induced oxidative stress through regulating mitochondrial dynamics in endothelial cells. Furthermore, the TyrRS-PARP1 signaling pathway was of significant importance in regulating mitochondrial fusion. Hence, these outcomes opened a new view of research regarding the potential protective role of RSV in treating AS.

## Conclusions

In conclusion, our results show that RSV regulates mitochondrial fusion through TyrRS-PARP1 signaling pathway, resulting in an attenuation of PA-induced endothelial oxidative stress. This finding may provide valuable clues in the search for new drugs that can be applied to mitochondria-targeting therapy in AS.

## Additional files

Additional file 1: Sequence information of siRNAs used in RNA knock down assay. Each siRNA used in the project is a pool of 3 different siRNA duplexes as listed in the table. (DOCX 13 kb)

Additional file 2: Sequence information of primers used in qRT-PCR. The sequence information of the primers used in qRT-PCR are listed in the table. (DOCX 13 kb)

Additional file 3: Antibodies used in the western blot experiments. The detail information of the antibodies used in western blot experiments are listed in the table. (DOCX 17 kb)

Additional file 4: The knock down effects of siRNAs of TyrRS and PARP1 in HUVECs. Cells were treated with siRNA of either control or target genes for 24 h, then harvested for RNA extraction. The mRNA of TyrRS and PARP1 were detected with qPCR and relative fold changes were displayed. *p < 0.05, **p < 0.01 vs. the control siRNA-treated group. (PDF 809 kb)

## Abbreviations

ACTB: β-actin
ANOVA: One-way analysis of variance
AS: Atherosclerosis
BSA: Bovine serum albumin
CCK-8: Cell Counting Kit–8
CVDs: Cardiovascular diseases
DCFH-DA: Dichloro-dihydro-fluorescein diacetate
DMSO: Dimethyl sulfoxide
eNOS: Endothelial nitric oxide synthase
FCM: Flow cytometry
GPx1: Glutathione peroxidase 1
HUVECs: Human umbilical vein endothelial cells
MDA: Malondialdehyde
MFN1: Mitofusin 1
MFN2: Mitofusion 2
MIM: Mitochondrial inner membrane
MMP: Mitochondria membrane potential
MOM: Mitochondrial outer membrane
NRF2: Nuclear factor-E2-related factor-2
OPA1: Optic atrophy type 1
PA: Palmitic acid
PARP1: Poly(ADP-ribose) polymerase 1
qRT-PCR: Quantitative real-time polymerase chain reaction
ROS: Reactive oxygen species
RSV: Resveratrol
SDS-PAGE: Sodium dodecyl sulfate-polyacrylamide gel electrophoresis
siRNA: Small interference RNA
SIRT1: NAD + −dependent histone/protein deacetylase sirtuin 1
SOD: Superoxide dismutase
TEM: Transmission electron microscopy
TyrRS: Tyrosyl transfer-RNA (tRNA) synthetase
