# Supplementary material for: Resveratrol attenuates oxidative injury in human umbilical vein endothelial cells through regulating mitochondrial fusion via TyrRS-PARP1 pathway
Source: Nutr Metab (Lond). 2019 Jan 30;16:9. doi: 10.1186/s12986-019-0338-7 (PMC6354417; doi:10.1186/s12986-019-0338-7)
Supplement: Supplementary file 1 — Sequence information of siRNAs used in RNA knock down assay. Each siRNA used in the project is a pool of 3 different siRNA duplexes as listed in the table. (DOCX 13 kb) [file 12986_2019_338_MOESM1_ESM.docx]

**Additional file 1:**

**Sequence information of siRNAs used in RNA knock down assay.**

| siRNA |  | Sequence (5′→3′orientation) |
| --- | --- | --- |
| TyrRS_A | Sense | CAAGAUUGAUCUCCUUGAUtt |
|  | Anti-sense | AUCAAGGAGAUCAAUCUUGtt |
| TyrRS_B | Sense | CCUGAAGAAUUCUGUUGAAtt |
|  | Anti-sense | UUCAACAGAAUUCUUCAGGtt |
| TyrRS_C | Sense | GUUGCAGGCUGACUUCAAAtt |
|  | Anti-sense | UUUGAAGUCAGCCUGCAACtt |
| PARP1_A | Sense | GAGUCAAGAGUGAAGGAAAtt |
|  | Anti-sense | UUUCCUUCACUCUUGACUCtt |
| PARP1_B | Sense | GGUAUCAACAAAUCUGAAAtt |
|  | Anti-sense | UUUCAGAUUUGUUGAUACCtt |
| PARP1_C | Sense | GCAACAAACUGGAACAGAUtt |
|  | Anti-sense | AUCUGUUCCAGUUUGUUGCtt |
