# Supplementary material for: Resveratrol attenuates oxidative injury in human umbilical vein endothelial cells through regulating mitochondrial fusion via TyrRS-PARP1 pathway
Source: Nutr Metab (Lond). 2019 Jan 30;16:9. doi: 10.1186/s12986-019-0338-7 (PMC6354417; doi:10.1186/s12986-019-0338-7)
Supplement: Supplementary file 2 — Sequence information of primers used in qRT-PCR. The sequence information of the primers used in qRT-PCR are listed in the table. (DOCX 13 kb) [file 12986_2019_338_MOESM2_ESM.docx]

**Additional file 2:**

**Sequence information of primers used in quantitative RT-PCR**

| Target gene | Primer sequence |
| --- | --- |
| TyrRS | F: 5’- GAGCTTGGCCGAGTTCTTTT-3’  R: 5’- GCAGACTTTCCCAGCTTTGC-3’ |
| PARP1 | F: 5’- GTGGATGGGTTCTCTGAGCT-3’  R: 5’- TACACCCCTTGCACGTACTT-3’ |
| ACTB | F: 5’- CTACAATGAGCTGCGTGTGG-3’  R: 5’- AAGGAAGGCTGGAAGAGTGC-3’ |
