# Supplementary material for: Resveratrol attenuates oxidative injury in human umbilical vein endothelial cells through regulating mitochondrial fusion via TyrRS-PARP1 pathway
Source: Nutr Metab (Lond). 2019 Jan 30;16:9. doi: 10.1186/s12986-019-0338-7 (PMC6354417; doi:10.1186/s12986-019-0338-7)
Supplement: Supplementary file 3 — Antibodies used in the western blot experiments. The detail information of the antibodies used in western blot experiments are listed in the table. (DOCX 17 kb) [file 12986_2019_338_MOESM3_ESM.docx]

**Additional file 3:**

**Antibodies used in the western blot experiments**

| Antibody | Dilution | Supplier |
| --- | --- | --- |
| TyrRS | 1:1000 | Santa cruz, US |
| PARP1 | 1:1000 | Abcam,UK |
| OPA1 | 1:1000 | Santa cruz, US |
| MFN1 | 1:1000 | Abcam, UK |
| MFN2 | 1:1000 | Abcam, UK |
| ACTB | 1:1000 | Cell Signaling Technology,US |
