# Supplementary figures and images for: Resveratrol attenuates oxidative injury in human umbilical vein endothelial cells through regulating mitochondrial fusion via TyrRS-PARP1 pathway
Source: Nutr Metab (Lond). 2019 Jan 30;16:9. doi: 10.1186/s12986-019-0338-7 (PMC6354417; doi:10.1186/s12986-019-0338-7)

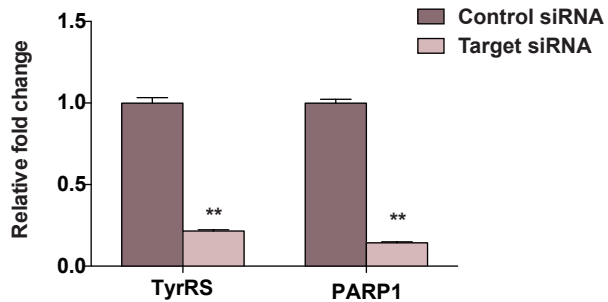

Supplement: Supplementary file 4 — The knock down effects of siRNAs of TyrRS and PARP1 in HUVECs. Cells were treated with siRNA of either control or target genes for 24 h, then harvested for RNA extraction. The mRNA of TyrRS and PARP1 were detected with qPCR and relative fold changes were displayed. *p < 0.05, **p < 0.01 vs. the control siRNA-treated group. (PDF 809 kb) [file 12986_2019_338_MOESM4_ESM.pdf]
